# Functional and splicing defect analysis of 23 *ACVRL1* mutations in a cohort of patients affected by Hereditary Hemorrhagic Telangiectasia

**DOI:** 10.1371/journal.pone.0132111

**Published:** 2015-07-15

**Authors:** Ferdos Alaa el Din, Sylvie Patri, Vincent Thoreau, Montserrat Rodriguez-Ballesteros, Eva Hamade, Sabine Bailly, Brigitte Gilbert-Dussardier, Raghida Abou Merhi, Alain Kitzis

**Affiliations:** 1 Genetics of rare diseases, University of Poitiers, Poitiers, France; 2 Department of Genetics, University Hospital of Poitiers, Poitiers, France; 3 Lebanese University Campus Hariri, Faculty of Science / EDST, Hadath, Lebanon; 4 INSERM U1036, CEA of Grenoble, Grenoble, France; 5 Competence Centre of Rendu-Osler, University Hospital of Poitiers, Poitiers, France; International Centre for Genetic Engineering and Biotechnology, ITALY

## Abstract

Hereditary Hemorrhagic Telangiectasia syndrome (HHT) or Rendu-Osler-Weber (ROW) syndrome is an autosomal dominant vascular disorder. Two most common forms of HHT, HHT1 and HHT2, have been linked to mutations in the endoglin (*ENG*) and activin receptor-like kinase 1 (*ACVRL1*or *ALK1*) genes respectively. This work was designed to examine the pathogenicity of 23 nucleotide variations in *ACVRL1* gene detected in more than 400 patients. Among them, 14 missense mutations and one intronic variant were novels, and 8 missense mutations were previously identified with questionable implication in HHT2. The functionality of missense mutations was analyzed in response to BMP9 (specific ligand of ALK1), the maturation of the protein products and their localization were analyzed by western blot and fluorescence microscopy. The splicing impairment of the intronic and of two missense mutations was examined by minigene assay. Functional analysis showed that 18 out of 22 missense mutations were defective. Splicing analysis revealed that one missense mutation (c.733A>G, p.Ile245Val) affects the splicing of the harboring exon 6. Similarly, the intronic mutation outside the consensus splicing sites (c.1048+5G>A in intron 7) was seen pathogenic by splicing study. Both mutations induce a frame shift creating a premature stop codon likely resulting in mRNA degradation by NMD surveillance mechanism. Our results confirm the haploinsufficiency model proposed for HHT2. The affected allele of *ACVRL1* induces mRNA degradation or the synthesis of a protein lacking the receptor activity. Furthermore, our data demonstrate that functional and splicing analyses together, represent two robust diagnostic tools to be used by geneticists confronted with novel or conflicted *ACVRL1* mutations.

## Introduction

Hereditary Hemorrhagic Telangiectasia (HHT) (ORPHA774, MIM # 187300) also known as Rendu-Osler-Weber disease is a vascular dysplasia syndrome, inherited as an autosomal dominant trait. It has an incidence of 1/8,000 persons being therefore a rare genetic disease [1–3]. The clinical symptoms characteristic of HHT are included in the Curaçao criteria [4]. Individuals with HHT initially present with epistaxis, telangiectases in mucocutaneous and gastrointestinal sites, arteriovenous malformations (AVMs) most commonly found in pulmonary, hepatic and cerebral circulations, and familial inheritance of a first-degree [5, 6].

HHT is a genetically heterogeneous disorder and has two most common forms HHT1 and HHT2 typically referring to the genes involved in each case [7]. Single mutations are detected in Endoglin (*ENG*; HHT1) (MIM # 131195) [8], Activin Receptor-Like Kinase 1 (*ACVRL1*/ALK1; HHT2) (MIM # 601284) [9, 10], or *MADH4*/SMAD4 (JHPT, a combined syndrome of juvenile polyposis and HHT) [11–13]. There are at least two other loci for HHT, HHT-3 and HHT-4, identified by linkage analysis mapped on chromosome 5q [4, 14] and chromosome 7p [15] respectively. A recent study revealed that mutations in bone morphogenetic protein 9, *BMP9* gene also known as *GDF2* (Growth Differentiation Factor 2), cause a vascular-anomaly syndrome with phenotypic overlap with HHT [16]. 80% to 90% of HHT cases present mutations in *ENG* or *ACVRL1* while the remaining cases are caused by mutations in *SMAD4* or in the other yet unknown genes [17, 18]. Over 800 different mutations in *ENG* and *ACVRL1* genes have been identified in patients with HHT1 and HHT2 respectively, pointing the wide allelic heterogeneity displayed by HHT. Among the point mutations described, missense mutations are mainly recorded (>46%) in HHT2 patients. Intronic and splice defect mutations are also noted but represents 8% of all mutations (http://arup.utah.edu/database/HHT/).

The protein products of *ENG*, *ACVRL1* and *MADH4* are receptors or signaling molecules of the TGFβ/BMPs pathway [19]. They are involved in the regulation of cell proliferation, differentiation, migration and extracellular matrix formation [20, 21]. In particular, they are expressed in endothelial cells [22, 23] that play a critical role for the proper development of the blood vessels [8, 10, 24]. *ACVRL1* encodes ALK1 which is a type I transmembrane serine/threonine kinase receptor and a partner for BMPR2 (type II transmembrane serine/threonine kinase receptor of the TGFβ pathway). *ENG* encodes Endoglin, a type I integral membrane glycoprotein that acts as a TGFβ type III receptor/co-receptor which collaborates with ALK1 to promote cell migration and proliferation [25–27]. Endoglin does not have a kinase activity but modulates ligand binding to its signaling receptors [28, 29]. Most TGFβ family ligands bind to an heterodimeric complexes of type I and type II serine/threonine kinase receptors [30, 31]. Upon ligand binding, the type II receptor phosphorylates and activates ALK1, one of the 7 type I receptors for TGFβ family members [28], which in turn phosphorylates a receptor-regulated Smad protein (Smad1, Smad5, or Smad8). This phosphorylated Smad dimerizes with a common partner, Smad4, and this complex translocates to the nucleus where it induces the transcriptional activation of specific genes [29]. In 2007, BMP9 and BMP10 were identified as the physiological ligands for ALK1 receptor, long been considered as an orphan receptor in endothelial cells [19, 32]. Subsequently, it was established that BMP9, but not BMP10, is the only active circulating member of the TGFβ family present in human adult serum capable of activating ALK1 and it could play a role as a regulator of endothelial quiescence [33].

In the present study, we analysed 23 mutations in *ACVRL1* gene that have been observed after the screening of more than 400 HHT patients. The discovery of BMP9 as the physiological ligand for ALK1 receptor in human serum [19, 33] enabled Nicolas Ricard *et al*. to establish a diagnostic tool based on the analysis of the BMP9 response through the BRE-Luciferase reporter gene activity to discriminate pathogenic from polymorphic *ACVRL1* mutations [1]. The first aim of the present work was to evaluate the functional significance of *ACVRL1* missense mutations found in our HHT2 patients in response to BMP9. Next, we aimed to elucidate *in cellulo* if the pathogenicity of an intronic mutation outside the standard splice sites, c.1048+5G>A at intron 7, was explained by a splicing impairment using the minigene approach. In addition, one missense mutation detected in exon 6, c.733A>G (p.Ile245Val), appeared functional after BMP9 stimulation while the patient is an index case. We hypothesized that this exonic substitution can disturb mRNA splicing. Minigene strategy was assessed and validated our assumption.

This work, based on functional analysis in response to BMP9 and splicing analysis using minigene approach confirms that the functional haploinsufficiency of the *ACVRL1* gene is the main pathophysiology underlying HHT2. Furthermore, the methodological approach has proved to be a useful tool to distinguish pathogenic *ACVRL1* mutations.

## Materials and Methods

### Patients

We reviewed data from 400 patients analysed in our laboratory between January 1^st^, 2005 and July 1^st^, 2014. Patients were diagnosed with HHT when they possessed at least three of the four Curaçao criteria: 1) an affected first degree family member; 2) recurrent nosebleeds; 3) multiple telangiectases along the mucocutaneous surface; and 4) AVMs in major organs [4].

Mutational analysis for the 4 genes, *ENG*, *ACVRL1*, *SMAD4* and *BMP9*, were carried out after written informed consent from the patients and under research protocols according to the declaration of Helsinki. Only missense variations of uncertain significance (VUS) were studied in this work. The authors did not have any interaction with patients and they used results of genetic screening without any identifying information for the patients.

### Sample Preparation and Genetic Screening

Genomic DNA was extracted from the peripheral EDTA-anticoagulated blood mononuclear cells (PBMCs) using the QIAamp DNA Mini Kit (Qiagen, Hilden, Germany). For mutational analysis, *ACVRL1*, *ENG* and *SMAD4* genes of probands were screened by direct DNA sequencing. Purified PCR amplicons encompassing exons and adjacent intron regions were sequenced on an ABI3130 automated sequencer (Applied biosystems, Foster City, CA, USA). The sequences were compared with wild type *ACVRL1* (GeneBank, NT_029419.11), *ENG* (GeneBank, NT_008470.18) and *SMAD4* (GeneBank, NT_010966.13).

### Plasmids

#### For the functional analysis

A wild-type (WT) construct of the *ACVRL1* cDNA cloned into the pcDNA3 vector was generated by Dr C. H. Heldin (Ludwig Institute for Cancer Research, Uppsala, Sweden) and kindly provided to us by Dr Sabine Bailly (University of Grenoble, Grenoble, France). This plasmid encodes WT-ALK1, HA-tagged at the C-terminus. This wild-type construct was used as a template to generate constructs containing the 22 missense point mutations through site-directed mutagenesis using the Stratagene QuikChange kit (Stratagene). A pcDNA3 empty vector was used as a control. The primer sequences used for site-directed mutagenesis are listed in S1A and S1B Table.

#### For minigene reporter

We used pSpliceExpress vector, a generous gift from Dr S. Stamm (University of Kentucky, USA). Three mutations were analysed in this study: the missense mutations c.733A>G (p.Ile245Val) in exon 6, c.1249A>T (p.Ile417Phe) in exon 9, and the intronic mutation c.1048+5G>A in intron 7. Patient DNA carrying each mutation was used to generate genomic fragments of *ACVRL1* including exon 6, exon 9 and exon 7 respectively with ±200bp of its upstream and downstream intronic sequences. These fragments were amplified by standard PCR using specific primers that contain the attB1 adapter F and attB2 adapter R recombination sites (listed in S2 Table), and Platinum Taq DNA Polymerase High Fidelity (Invitrogen). PCR products were then purified using QIAquick PCR Purification Kit Protocol (QIAGEN). Next, 5–150 ng of the attB-PCR product was mixed to 150 ng of pSpliceExpress vector in 10 μl of recombination reaction, to which was added 2 μl of the BP Clonase II enzyme mix and sufficient volume of TE buffer, pH 8, to complete the total reaction volume. The reaction was incubated at 25°C for 1 hour. Then 1 μl of proteinase K (2 mg/ml; Invitrogen) was added to inactivate the enzymes. The samples were subsequently incubated at 37°C for 10 minutes to terminate the reaction and used later to transform competent E. coli (Invitrogen).

### Transformation

1 μl of each BP reaction was transformed into 50 μl of One Shot TOP10 Chemically Competent E. coli (Invitrogen) according to the manufacturer’s instructions. This E. coli strain does not contain the F′ episome. This episome contains ccdA gene, which prevents negative selection with the ccdB gene. Later, the transformed bacteria were plated on Ampicillin-supplemented LB plates and incubated at 37°C overnight. Single colonies were inoculated in 5 ml of LB medium overnight and their plasmid DNA was extracted by QIAprep Spin Miniprep Kit (QIAGEN). Subsequently, KpnI restriction digestion was performed to confirm the presence of the desired insert. The remaining plasmid extract was treated with RNAase (10 mg/ml) for 1h at 37°C; then re-extracted with a classical phenol chloroform protocol. Minigenes carrying WT or variant alleles were verified by sequencing.

### Functional assay

The effects of ALK1 missense mutations on its activity were investigated by the analysis of the BMP9 response assay established by Nicolas Ricard *et al*. [1]. The reporter plasmid pGL3(BRE)2-luc encoding firefly luciferase downstream of a BMP response element [34] was kindly provided by Dr P. Ten Dijke (Leiden University Medical Center, Leiden, The Netherlands). The pRL-TK-luc plasmid encoding renilla luciferase downstream of the thymidine kinase promoter was kindly provided by Dr Sabine BAILLY. NIH-3T3 cells maintained in Dulbecco modified Eagle medium, 4.5 g/L glucose (Invitrogen) supplemented with 10% fetal bovine serum (Biowest) and 1% of Penicillin/Streptomycin (Pen Strep; gibco), were transfected in Opti-MEM (Invitrogen) using Lipofectamine 2000 (Invitrogen) with 0.1 μg of pGL3(BRE)2-luc, 0.02 μg of pRL-TK-luc, and 0.5 μg of plasmids encoding WT-ALK1 or the different mutants. Four hours after transfection, cells were treated with or without recombinant BMP9 (100 pg/mL; R&D Systems) for 15 hours. Firefly and renilla luciferase activities were measured sequentially with the Dual-Luciferase reporter assay (Promega) using LUMINOMETER GloMax 20/20 (Promega).

### Transfection and RT-PCR

The positive clones carrying the DNA insert (WT and variants alleles) were cultured in 50ml of LB medium overnight and plasmids were extracted using DNeasy Blood and Tissue Kit (QIAGEN). Then the minigene constructs were transfected in HeLa cells for 48 hours using Lipofectamine 2000 (Invitrogen). 48h later, total RNA was extracted using RNABLE (Eurobio) according to the manufacturer’s instructions. The RNA samples were quantified by spectrophotometry. The first strand cDNA was generated by reverse transcription (RT) reaction using M-MLV Reverse Transcription Kit (Promega). The RT components in a total volume of 25 μl contained 5 μl of 5X reaction buffer, 1 μl of 25 mM dNTPs, 1 μl of Oligo(dT)_15_ Primers (500 μg/ml), 2 μl of M-MLV reverse transcriptase (200 units), 1 μl of RNase inhibitor (1 unit), 1 μg of RNA and nuclease free ddH_2_O. RT was done by incubation at 37°C for 1 hour followed by enzyme inactivation at 100°C for 2 minutes. PCR was performed using AmpliTaq Gold DNA Polymerase with Gold Buffer and MgCl_2_ (Applied biosystems) according to the supplier’s protocol and the PCR products were analyzed on Agarose gel (2%) or TapStation (Agilent). pSpliceExpress vector contains two constitutively expressed insulin exons, which are spliced together and serve as a positive control when the vector generates mRNA species that reflect the splicing pattern of the subcloned genomic fragments. RT-PCR products were analyzed further by sequencing.

### Sequencing

Clones obtained after transformation and cDNAs from RT were amplified by appropriate PCRs. PCR products were treated with illustra ExoProStar 1-step (GE Healthcare) to remove any remaining primers and dNTPs at 37°C for 15 minutes then 80°C for 15 minutes to inactivate the enzyme. Purified PCR products were subsequently submitted to automated sequence analysis on ABI Prism 3130 Genetic Analyser using the ABI PRISM Big Dye terminator Cycle Sequencing kit (Applied Biosystems) with targeted primers.

### Western blot analysis

HeLa cells were transfected using Lipofectamine 2000 (Invitrogen) following the supplier’s instructions with 2μg of plasmids encoding WT-ALK1 or different mutants. After 48 hours incubation, cells were washed twice with PBS (Phosphate-Buffered Saline) and lysed in radioimmunoprecipitation assay (RIPA) buffer (50 mM Tris-HCl (pH 7.5), 1 mM EDTA, 100 mM NaCl, 1% Triton X-100) with a cocktail of protease inhibitors (Complete, Roche). Protein lysates were separated by sodium dodecyl sulfate polyacrylamide gel electrophoresis (SDS-PAGE; 10%) and analyzed by immunoblotting with anti-HA (H3663; Sigma- Aldrich) and anti-βactin (A5316; SIGMA) antibodies.

### Immunofluorescence

HeLa cells grown on glass coverslips were washed with PBS, fixed with 3.7% paraformaldehyde for 5–10 min. Fixed cells were then permeabilized in PBS-0.5% BSA- 0.1% Triton solution for 5–10 min and incubated in blocking solution (PBS-0.5%BSA) for 60 min at room temperature. The permeabilized cells were then incubated for 1 h at room temperature with mouse monoclonal anti-HA antibodies and rabbit polyclonal anti-calnexin antibodies (SPA-860; Stressgen biotechnologies) diluted in blocking buffer. After PBS washing, the cells were incubated with fluorescent secondary antibodies, Fluoprobes 488 Donkey anti Mouse IGG (INTERCHIM) and Rhodamine Red_X_conjugated Donkey anti-Rabbit IgG (Jackson ImmunoResearch) for 1 h at room temperature then washed several times with PBS and mounted in immunofluor medium (ICN Biomedicals). Labeled cells were analysed with a spectral confocal microscope, Olympus FV1000.

### Splice site prediction

Three different *in-silico* splice site prediction tools were used to predict a potential splicing effect: Human Splicing Finder (HSF) (Desmet et al., 2009), GeneSplicer (Pertea et al., 2001) and NetGene2 (Brunak et al., 1991).

## Results

### Studied mutations

**Fig 1:**
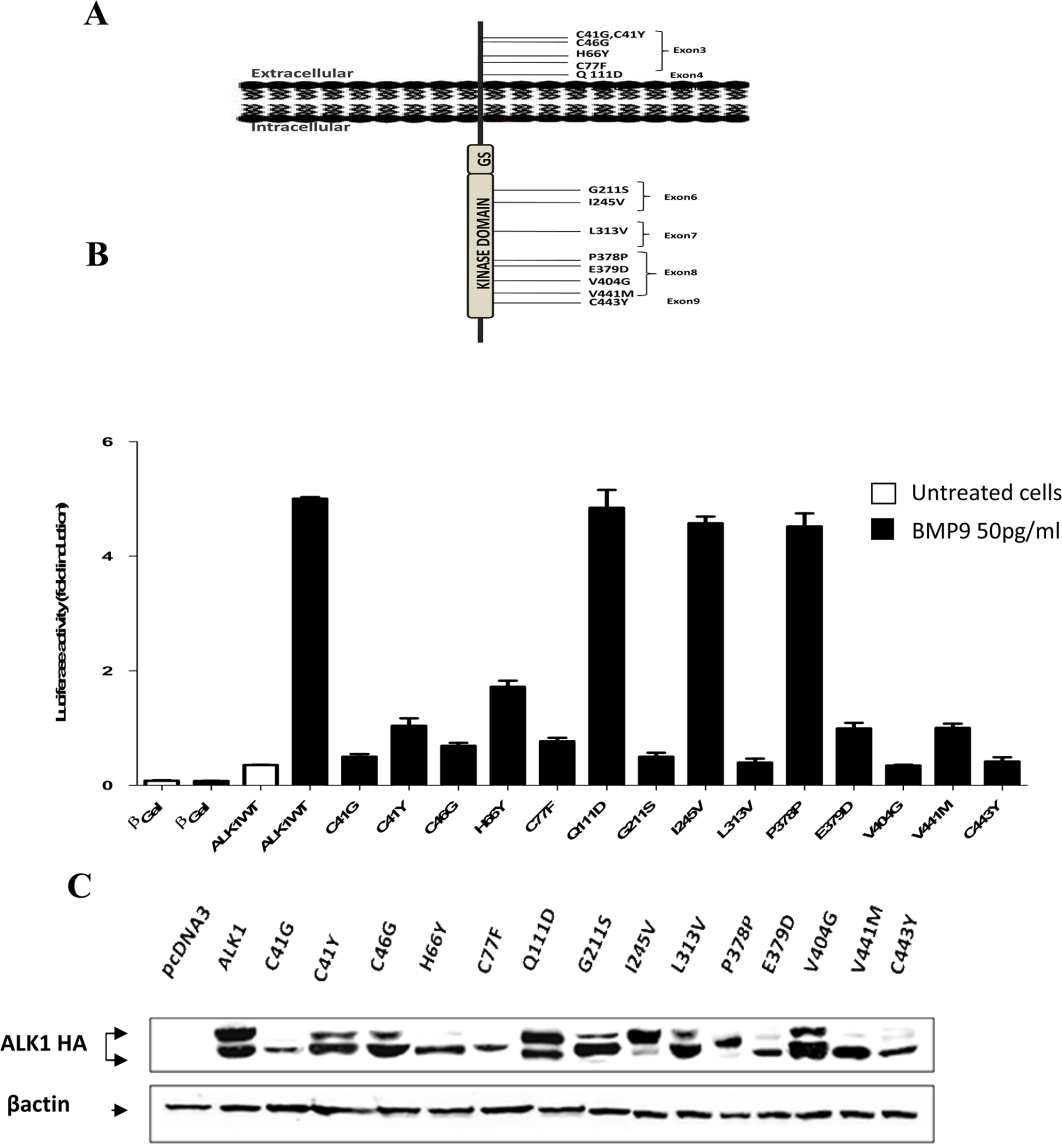
BMP9 response and western blot analysis of ALK1 novel mutants. (A) Schematic localization of the 14 novel mutations on the ALK1 protein. The exons in which the nucleic acid substitutions occur and their resulting amino acid substitutions are indicated. The mutations are distributed in the extracellular and the kinase domain of the ALK1 receptor. (B) Functional analysis of the ALK1 protein variants was performed by measuring luciferase reporter activity after BMP9 stimulation, as explained in materials and methods section. Results are expressed as fold induction over the value obtained for each ALK1 mutant in the absence of BMP9. Data are mean ± SD of 3 independent experiments. (C) Western blot analysis of ALK1 protein variants was performed after transient transfection of HeLa cells by expression vectors encoding a C-terminally HA-tagged version of either WT-ALK1 or the different novel ALK1 mutants. Transfection with an empty pcDNA3 vector was used as control. After 48 hours, cells were lysed and 50 μg of cell lysates were resolved by 10% SDS-PAGE and immunoblotted with antibodies against HA or against βactin as a loading control.

**Fig 2:**
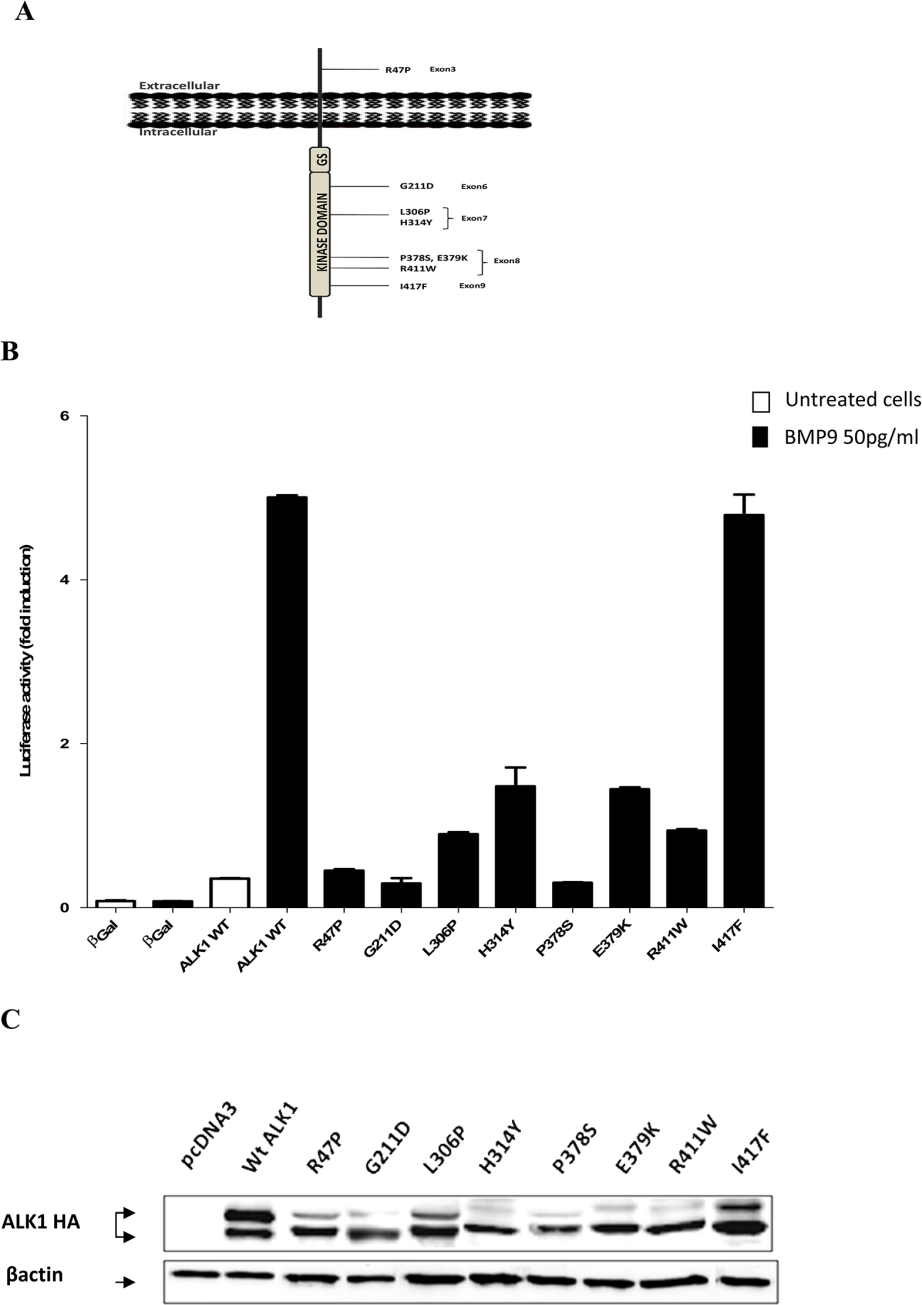
BMP9 response and western blot analysis of ALK1 known mutants. (A) Schematic localization of the 8 known mutations on the ALK1 protein. The exons in which the nucleic acid substitutions occur and their resulting amino acid substitutions are indicated. The mutations are distributed in the extracellular and the kinase domain of the ALK1 receptor. (B) Functional analysis of the ALK1 protein variants was performed by measuring luciferase reporter activity after BMP9 stimulation, as explained in materials and methods section. Results are expressed as fold induction over the value obtained for each ALK1 mutant in the absence of BMP9Data are mean ± SD of 3 independent experiments.(C) Western blot analysis of ALK1 protein variant was performed after transient transfection of HeLa cells by expression vectors encoding a C-terminally HA-tagged version of either Wt-ALK1 or the different novel ALK1 mutants. Transfection with an empty pcDNA3 vector was used as a control. After 48 hours, cells were lysed and 50 μg proteins of cell lysates were resolved by 10% SDS-PAGE and immunoblotted with antibodies against HA or against βactin as a loading control.

All probands of the present study have been diagnosed with clinical features of the HHT disorder including recurrent nosebleeds, mucocutaneous telangiectasia, and arteriovenous malformations in lung, liver and central nervous system. Mutational analysis of the entire *ACVRL1* coding sequences in our group of more than 400 patients enabled us to detect several mutations. We focused our study on 23 mutations: 22 missense mutations and one intronic variant. Fourteen out of the 22 missense mutations had never been reported before in HHT2 patients and eight had been previously described with uncertain pathogenicity (http://arup.utah.edu/database/HHT/). Among the fourteen novel mutations, 6 were in the extracellular domain (p.Cys41Gly, p.Cys41Tyr, p.Cys46Gly, p.His66Tyr, p.Cys77Phe, and p.Glu111Asp) and 8 were localized in the intracellular kinase domain (p.Gly211Ser, p.Ile245Val, p.Leu313Val, p.Pro378Pro, p.Glu379Asp, p.Val404Gly, p.Val441Met, and p.Cys443Tyr) (Fig 1A). The silent nucleotide substitution (c.1134C>T; p.Pro378Pro) is a novel polymorphism and it was included in this study as an internal control. One of the known mutations was found in the extracellular domain (p.Arg47Pro) and the seven remaining mutations were in the intracellular domain (p.Gly211Asp, p.Leu306Pro, p.His314Tyr, p.Pro378Ser, p.Glu379Lys, p.Arg411Trp, and p.Ile417Phe) (Fig 2A). The intronic mutation, c.1048+5G>A, was located at the +5 of the canonical splice site within intron 7 of *ACVRL1*.

### Functional assay

To study the pathogenicity of our HHT2-related ALK1 mutations, we used site-directed mutagenesis to generate 22 mutants reproducing either known or novel human mutations found in our patients. First, we investigated the impact of these mutations on the ALK1 receptor activity in response to BMP9, using luciferase reporter controlled by Smad 1/5-transcriptional responsive element to BMP9, pGL3 (BRE)_2_-luciferase [34]. For this, NIH3T3 cells, that do not express endogenously ALK1, were transfected with ALK1 mutants and pGL3 (BRE)_2_-luciferase plasmid vectors. Four hours after transfection, cells were stimulated with BMP9 (50 pg/ml: EC_50_ [33]) for 15 hours. BMP9 induced a 5-fold induction of the luciferase activity in the cells transfected with WT-ALK1 (Figs 1B and 2A). Most ALK1 novel mutants did not respond to BMP9 stimulation as shown in Fig 1B except p.Glu111Asp, p.Ile245Val, and p.Pro378Pro which responded to BMP9 similarly to WT-ALK1 (4.9 and 4.8-fold induction). Similarly, in the series of known mutations, the addition of BMP9 stimulated the BMP Response Element (BRE) in the case of the WT-ALK1 but not with ALK1 mutants except p.Ile417Phe (4.9 fold induction) which was equal to the WT-ALK1 response (Fig 2B).

### Western blot analysis

In order to verify the results of the functional assay in response to BMP9, we observed whether these mutations affect the protein maturation. HeLa cells were used to transiently express ALK1, including WT and mutant constructs. 48 hours later, cells were lysed and proteins were analyzed by western blot using anti-HA and anti-actin antibodies. Immunoblotting results showed that for the WT-ALK1, two protein bands were detected, the upper and the lower bands representing respectively the mature ALK1 protein with complex N-glycans and the precursor immature form. The WT-ALK1 has slightly intense upper band compared to the lower band. However the majority of the mutants, (p.Cys41Gly, p.Cys41Tyr, p.Cys46Gly, p.His66Tyr, p.Cys77Phe, p.Gly211Ser, p.Leu313Val, p.Glu379Asp, p.Val404Gly, p.Val441Met and p.Cys443Tyr) from the novel mutations (Fig 1C) and (p.Arg47Pro, p.Gly211Asp, p.Leu306Prol, p.His314Tyr, p.Pro378Ser, p.Glu379Lys and p.Arg411Trp) among the previously described mutations (Fig 2C), have a predominant lower immature band. This aspect reflects an impaired folding affecting the processing of the proteins and probably their retention in a pre-Golgi compartment. The p.Glu111Asp, p.Ile245Val, and p.Ile417Phe mutants have an aspect equivalent to WT-ALK1.

### Cellular localization

**Fig 3:**
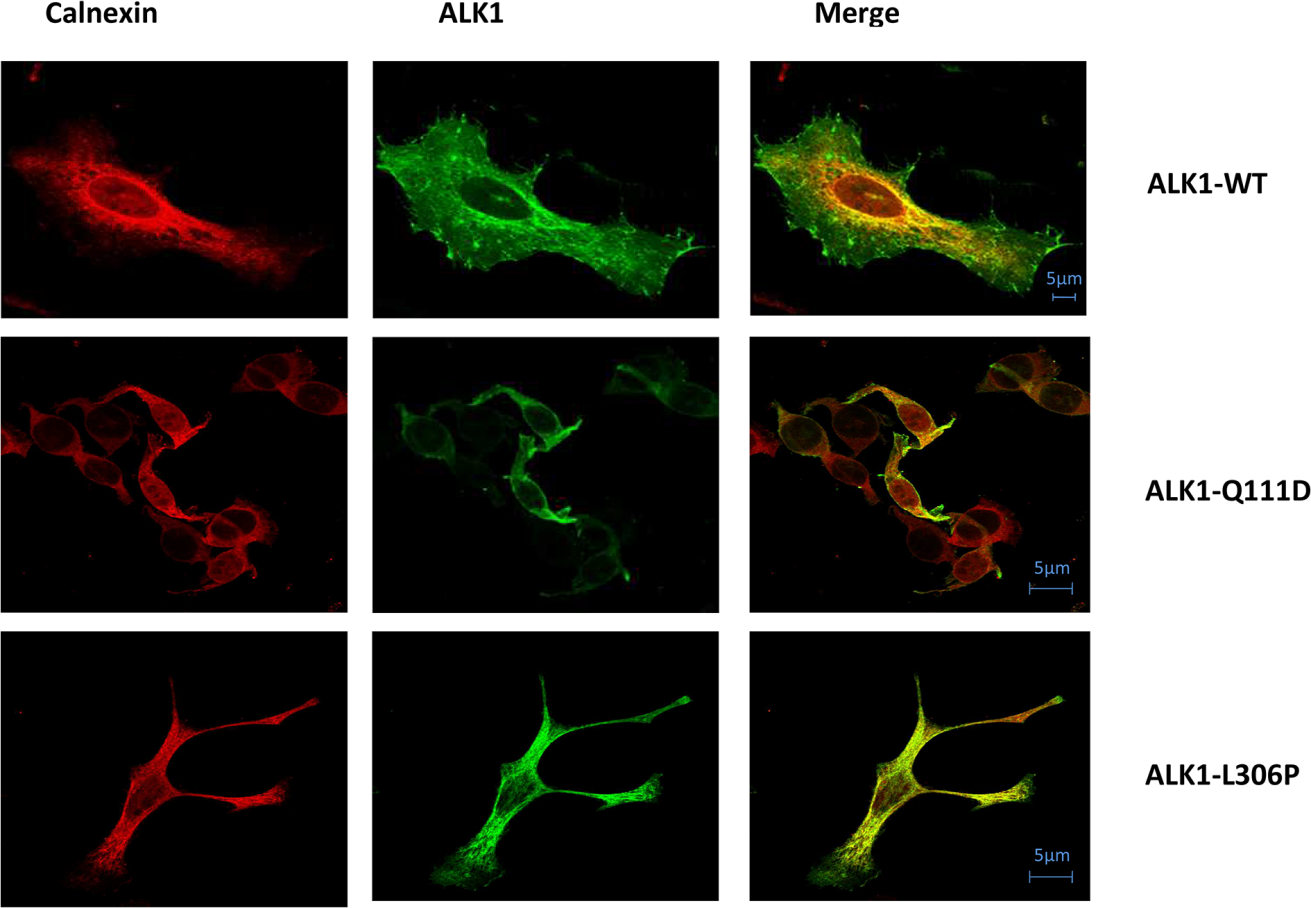
Subcellular localization of ALK1 protein variants. HeLa cells were transiently transfected with the C-terminally HA-tagged ALK1 plasmids. Subsequently the permeabilized cells were co-stained with mouse monoclonal anti- HA antibody and with rabbit polyclonal anti- calnexin antibody and then processed for fluorescence confocal microscopy. In represented photos, the mutant Q111D reached the cell surface similarly to the ALK1-Wt. However the mutant L306P co-localized with the calnexin in the ER network was predominant (individual photos displaying each studied mutant ALK1 protein are presente in figure S1). Bars = 5μm.

Subsequently, to indicate if our studied missense substitutions impair the trafficking of the mutant ALK1 receptors, HeLa cells expressing HA-tagged ALK1-wild type or ALK1 mutants were labeled with the mouse anti-HA monoclonal antibody and a rabbit anti-calnexin polyclonal antibody. Calnexin is a chaperone that acts to retain unfolded or unassembled N-linked glycoproteins in the Endoplasmic Reticulum (ER). The subcellular localization was then examined by immunofluorescence analysis under confocal laser scanning microscopy. Double labeling revealed that there was no significant co-localization of the wild-type ALK1 protein with calnexin. Wild-type ALK1 was localized predominantly on the plasma membrane with additional staining throughout the ER network, confirming that wild-type ALK1 is transported out of the ER system and is addressed to the plasma membrane. *A contrario*, all ALK1 mutant proteins, with the exceptions of p.Ile417Phe, p.Glu111Asp, p.Ile245Val, and p.Pro378Pro, were observed to be distributed intracellularly in a perinuclear and reticular pattern typical of the ER network in co-localization with calnexin, suggesting that expressed mutant proteins are mostly retained in the ER (Fig 3 and S1 Fig). Some mutant proteins such as p.Cys41Gly, p.Cys41Tyr, p.Leu313Val, p.Arg411Trp, and p.Val404Gly, showed a membrane additional staining but we observed that they were preferentially targeted to the ER compartment rather than transported to the cell surface (S1 Fig).

### Splicing analysis

**Fig 4:**
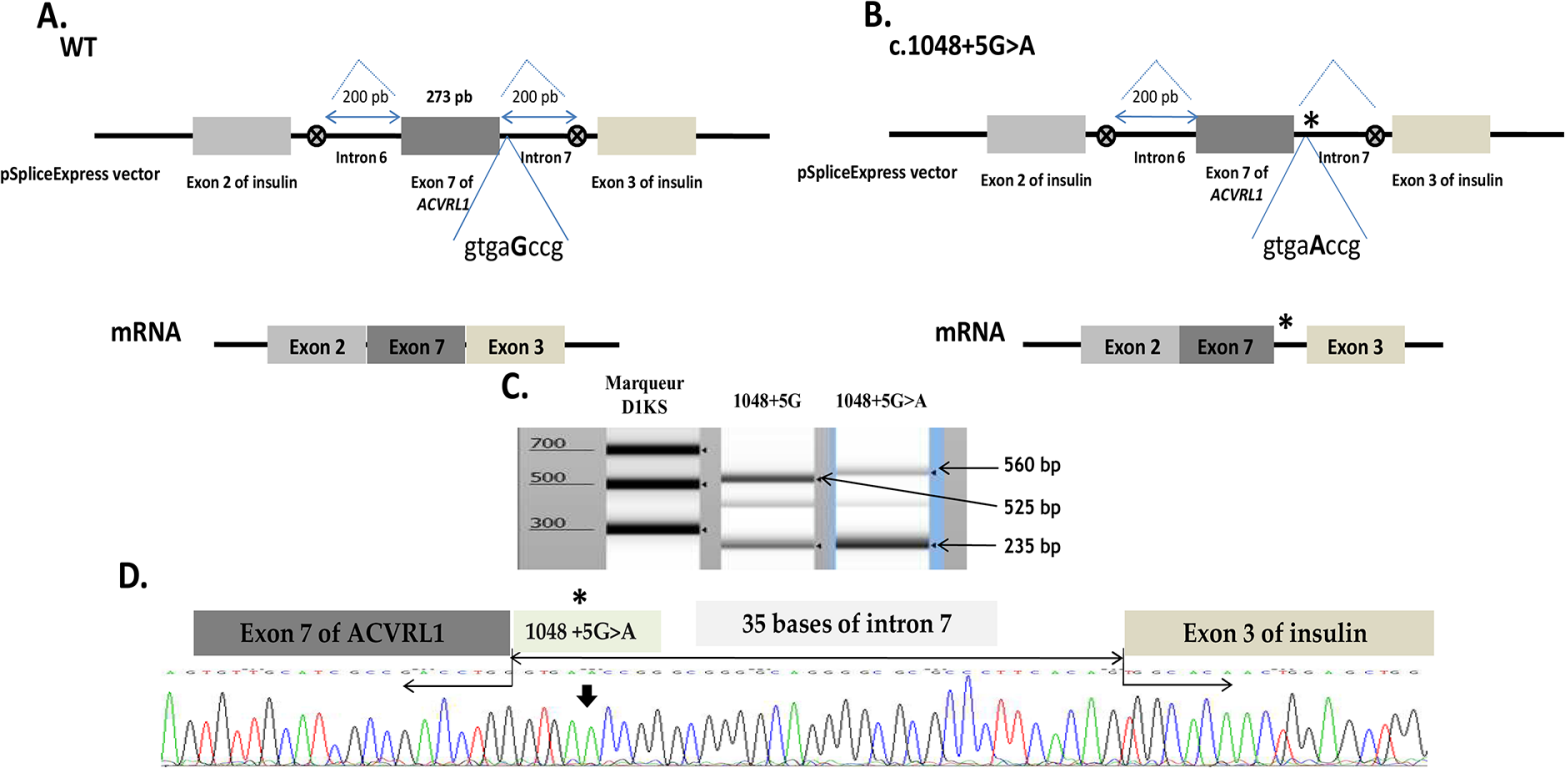
Splicing defect induced by the c.1048+5G>A mutation. (**A, B**) Schematic construction of pSpliceExpress reporter minigene containing exon 7, Wt and mutant, and their corresponding mRNAs. The position of the c.1048+5G>A mutation is indicated with a star (on the right). The first eight nucleotides downstream of the exon-intron junction are indicated. Abnormal longer transcript generated by the new donor splice-site mutation c.1048+5G>A is shown. (**C**) Electrophoresis of RT-PCR products obtained after transfection in HeLa cells of normal allele, c.1048+5G (lane 2) and mutant allele, c.1048+5G>A (lane 3) revealed on TapStation (Agilent). Samples were amplified using oligonucleotides complementary to exons 2 and 3 of insulin. The uppermost band in lane 2 of 525 bp corresponds to the normally spliced wt mRNA of the exon 7 with exons 2 and 3 of insulin. The uppermost band in lane 3 of about 560 bp correponds to the aberrant transcript resulted from a splicing failure and inclusion of an intronic part in the mRNA. The lower band in lane 2 and 3 corresponds to the band containing the exons of insulin alone without the exon 7 of ACVRL1. (**D**) Sequencing analysis of the longer transcript resulting from the mutant construct including 35 bp of the intron 7.

To determine the impact on splicing of the c.1048+5G>A mutation in intron 7, we generated a minigene reporter by cloning PCR-amplified genomic exon 7 and ± 200bp of adjacent introns into pSpliceExpress vector, using a single recombinant reaction based on the gateway system [35]. After transfection in HeLa cells, the splicing products were assayed by RT-PCR to investigate the consequences of c.1048+5G>A on splicing. As reported in Fig 4C, the RT-PCR shows two distinct mRNA species in either the c.1048+5G>A and the WT constructs with a difference in the size of the upper band (compare lane 2 with lane 3 in Fig 4C). The upper band of about 525bp in lane 2 corresponds to normally spliced mRNA product whereas the 560bp band in lane 3 represents an aberrant transcript. The lower band of 235bp was the same in both cases and represents the insulin exons, which are present in the pSpliceExpress and are constitutively spliced together in most cases to serve as an internal positive control. Sequencing analysis of the RT-PCR fragments in Fig 4D, revealed that the longer transcript resulted from the retention of 35bp of the intron 7 due to the activation of a novel donor splice site, inducing a frame shift with a Premature Termination Codon (PTC) in the exon 8, predicting that the resulting mRNA is a substrate for Nonsense Mediated Decay (NMD).

**Fig 5:**
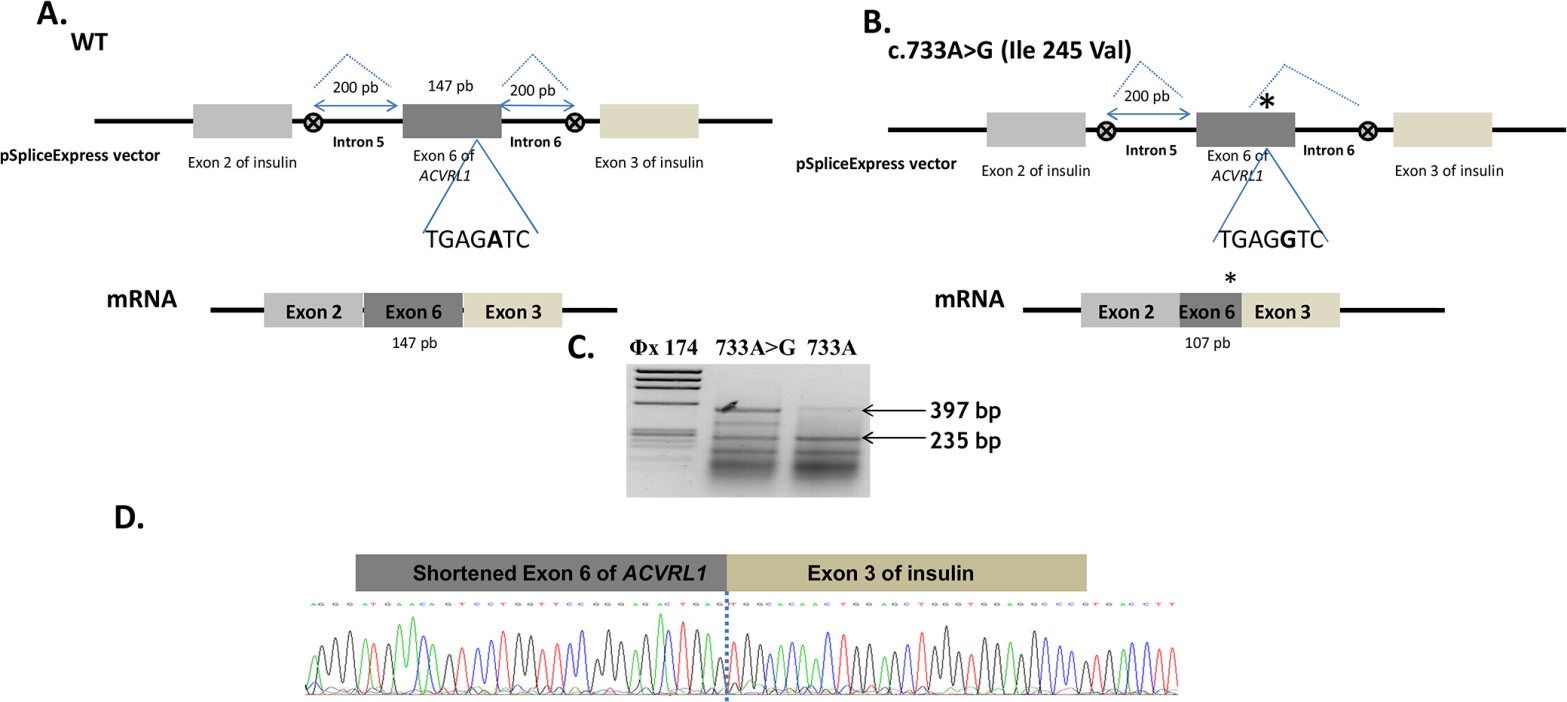
Splicing defect induced by the c.733A>G (Ile245Val) mutation. (**A, B**) Schematic construction of pSpliceExpress reporter minigene containing exon 6, Wt and mutant, and their corresponding mRNAs. The position of the c.733A>G (Ile245Val) mutation is indicated with a star (on the right). The seven nucleotides around the mutation are indicated. The c.733A>G mutation induces the creation of a new donor splice site and the loss of the 3’ end of exon 6. (**C**) Electrophoresis of RT-PCR products obtained after transfection in HeLa cells of c.733A>G, mutant and normal alleles, revealed on 2% agarose gel. Samples were amplified using oligonucleotides complementary to exons 2 and 3 of insulin gene. The uppermost band of 397 bp in lane 3 corresponds to the normally spliced wt mRNA of the exon 6 with exons 2 and 3 of insulin. The band of about 357 bp in lane 2 correponds to the aberrant transcript resulting from a splicing failure and loss of an exonic part in the mRNA. The lower band in lane 2 and 3 corresponds to the fragment containing the exons of insulin alone without the exon 6 of ACVRL1. (**D**) Sequencing analysis of the short transcript that resulted from the mutant construct transfection, missing 40 bp of the exon 6.

**Fig 6:**
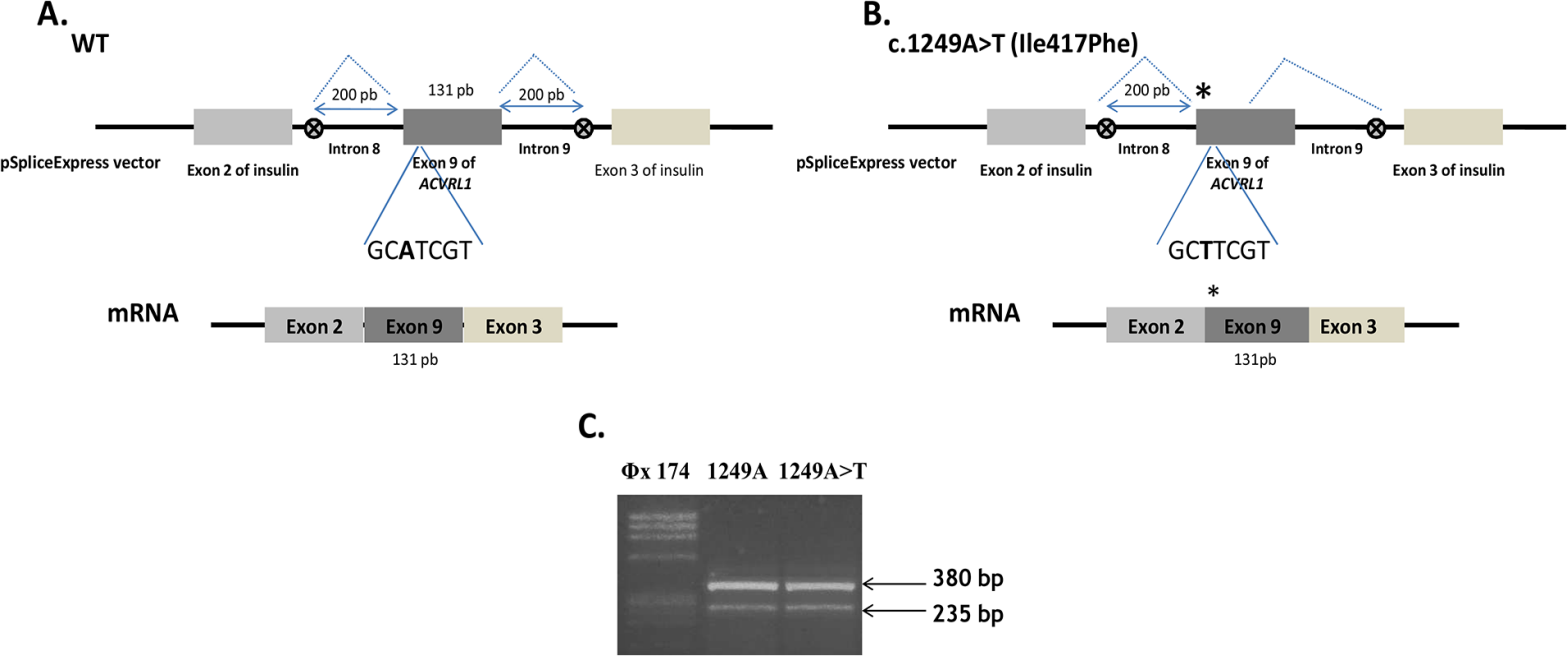
Normal splicing resulting from the c.1249A>T (Ile417Phe) mutation. (**A, B**) Schematic construction of pSpliceExpress reporter minigene containing exon 9, Wt and mutant, and their transcripted mRNAs. The position of the c.1249A>T (Ile417Phe) mutation is indicated with a star (on the right). The seven nucleotides around the mutation are indicated. A normal transcript generated by the mutant allele is shown. (**C**) Electrophoresis of RT-PCR products obtained after transfection in HeLa cells of normal allele, c.1249A (lane 2) and mutant allele, c.1249A>T (lane 3) revealed on 2% agarose gel. Samples were amplified using oligonucleotides complementary to exons 2 and 3 of insulin. The higher band in both cases of 380 bp corresponds to the normally spliced wt mRNA of the exon 9 with exons 2 and 3 of insulin (sequence not shown).

The splicing reporter minigene approach was applied to the missense mutations that responded to BMP9 stimulation in order to uncover their molecular mechanism. We tested c.733G>A (p.Ile245Val) in exon 6 and c.1249A>T (p.Ile417Phe) in exon 9. The exons 6 and 9 and their ≈200bp flanking intronic sequences were amplified by PCR using specific primers. The hybrid constructs were then transfected into HeLa cells and the RT-PCR were performed. In the case of c.733A>G (p.Ile245Val) (Fig 5C), we observed three species of RNA compared to two products for the WT allele (compare lane 2 with lane 3). The upper band obtained with the normal exon splicing, around of 400bp, was slightly higher than the band obtained with the mutant exon and we found another band in lane 2 between the upper band and the lower band of insulin exons. Sequencing analysis of the RT-PCR fragment in Fig 5D, revealed that the short fragment resulted from the c.733A>G allele having lost 40 bp from the exon 6 due to the activation of a novel acceptor splice site, inducing a frame shift with a PTC in the exon 7 predicting a substrate of NMD. Unfortunately, the middle band was impossible to sequence due to its low intensity. In the case of the c.1249A>T substitution in exon 9, the bands of the RT-PCR products were the same for the WT construct as well as for the mutant construct. Two bands were observed on gel, one of 235bp corresponding to the insulin exons and one heavier band of 380bp (Fig 6C). Sequencing analysis of this last band revealed that it corresponds to the complete exon 9 in both cases (sequence not represented).

In summary, our results revealed that the missense mutations have mostly a critical effect on the protein structure, totally spoiling or reducing in part, the protein function. However, c.733A>G, p.Ile245Val has an upstream effect and contributes to a radical inhibition of the mRNA translation by disturbing the splicing mechanism. Furthermore, our data demonstrate that an intronic mutation placed nearby the classical splice sites can deregulate the normal predicted splicing event and promote the activation of novel regulatory cis-elements. In our case, c.1048+5G>A reduces the normal splicing site efficiency and therefore activates a cryptic donor splice site 35bp upstream of the 3’end of the intron 7, inducing a frame-shift with a PTC in the exon 8. Taken together, our findings suggest that the analysis of the BMP9 response and the generation of the minigene reporter constructs are two particularly useful diagnostic tools and both constitute a solid approach to discriminate pathogenic from polymorphic *ACVRL1* mutations.

## Discussion

HHT is an autosomal dominant disease affecting approximately 1.2 million people worldwide [1]. This syndrome involves mainly mutations in the gene encoding Endoglin (*ENG*) related to HHT1, or in the gene encoding ALK1 (*ACVRL1*), in the case of HHT2. Patients with *ENG* mutations were excluded from this study. Most *ENG* mutations found in our laboratory were frameshift and result in a loss of the mRNA; therefore there was no doubt about their pathogenicity. However, a cellular assay was recently published in order to study the functional significance of endoglin missence mutations, based on the ability of endoglin to enhance ALK1 response to bone morphogenetic protein 9 (BMP9) [36].

For *ACVRL1*, a large spectrum of mutations within its locus has been identified as being responsible for HHT2 (http://www.hhtmutation.org). Mutations in this gene are distributed over the entire coding sequence and can also be detected in intronic splice sites. In the present work, we report the molecular characterization of 23 selected mutations of *ACVRL1* gene. We performed functional and/or splicing analysis to assess the morbidity of these mutations.

The functional assay in response to BMP9 was useful to evaluate the pathologic significance of the missense mutations. This functional test of the ALK1 receptors was modeled to replace the TGF-β response analyzed in previous studies [37, 38]. BMP9 is expected to activate the canonical bone morphogenic protein (BMP) signaling pathway which is mediated via the ALK1/2/3/6 type I receptors that phosphorylate Smad 1, Smad 5 and Smad 8 [39]. These intracellular mediators are in turn able to activate BRE (BMP Responsive Element) identified in Id1 promoter [34]. The splicing reporter minigene is the most common technique used to analyze the regulation of an alternative splicing and it provides an avenue to study splice site selection *in cellulo* [40]. In opposition to the traditional laborious construction of splicing reporter minigene using restriction enzyme digestion [41], the facility of the pSpliceExpress system, permits its use in medically relevant application in our laboratory. Combinating these two techniques, we developed a strategy for routine diagnosis, in attempt to analyze a large panel of ALK1 mutants detected by genomic DNA screening. In our cohort of 400 HHT2 patients, we detected 23 mutations, 22 were of missense type and one was intronic. Out of the 22 missense mutations, 8 were already published and 14 had never been described in databases.

Firstly, our functional test results discriminate 18 non-functional mutants versus 4 functional mutants (Table 1). Performing western blot was necessary to observe the maturation of mutant proteins. We noticed a difference in the band intensity between the WT-ALK1 and the 18 defective receptors. While the mature higher band was predominant compared with the immature lower band in the case of the WT-ALK1, this band was thin compared to a large lower band in the case of ALK1 mutants and in some cases absent as for p.Cys41Gly, p.His66Tyr, and p.Cys77Phe. Western blot and functional assay results were next confirmed with immunofluorescence staining. Confocal microscopy showed clearly that the wild type ALK1 is predominantly present at the plasma membrane. ALK1-WT reveals a partial co-localization with the ER marker (calnexin) indicating that newly synthesized ALK1 is present in the ER for some time to allow its folding. In opposite, the 18 non-functional receptors were retained in the ER compartment and the others were targeted to the cell surface similarly to the WT-ALK1. Therefore, regarding the 18 pathogenic mutations, our data link the reduced mature band with the intracellular localization in accordance with defective response obtained by the functional analysis. Moreover, the co-localization of the pathological mutants with the ER resident marker (calnexin) reflects their defective trafficking and subsequently their retention in the ER by the quality control mechanism. This eventually should lead to the degradation of these misfolded proteins by the ER-Associated protein Degradation (ERAD) system contributing to the disease. This scenario is consistent with the cellular mechanism described for HHT [42, 43]. To further confirm this result, the N-glycosylation assay of the immunoprecipitated proteins can be performed.

**Table 1:** Functional significance of ACVRL1 mutations. Previously known mutations are indicated in bold. *Patients with another described mutation in ENG.

Nucleotide change	Amino acid change	Exon	Location of mutation	Conflicting mutation *	BMP9 response	Expression	Localization
c.121T>G	p.Cys41Gly (C41G)	3	Extracellular	-	No	Yes	Intracellular
c.122G>A	p.Cys41Tyr (C41Y)	3	Extracellular	-	No	Yes	Intracellular
c.136T>G	p.Cys46Gly (C46G)	3	Extracellular	-	No	Yes	Intracellular
**c.140G>C**	**p.Arg47Pro (R47P)**	**3**	**Extracellular**	**-**	**No**	**Yes**	**Intracellular**
c.196C>T	p.His66Tyr (H66Y)	3	Extracellular	-	No	Yes	Intracellular
c.230G>T	p.Cys77Phe (C77F)	3	Extracellular	-	No	Yes	Intracellular
c.333G>C	p.Glu111Asp (Q111D)	4	Extracellular	yes	Yes	Yes	Cell surface
c.631G>A	p.Gly211Ser (G211S)	6	Kinase domain	-	No	Yes	Intracellular
**c.632G>A**	**p.Gly211Asp (G211D)**	**6**	**Kinase domain**	**-**	**No**	**Yes**	**Intracellular**
c.733A>G	p.Ile245Val (I245V)	6	Kinase domain	-	Yes	Yes	Cell surface
**c.916G>C**	**p.Ala306Pro (L306P)**	**7**	**Kinase domain**	**-**	**No**	**Yes**	**Intracellular**
c.937C>G	p.Ala313Val (L313V)	7	Kinase domain	-	No	Yes	Intracellular
**c.940C>T**	**p.His314Tyr (H314Y)**	**7**	**Kinase domain**	**-**	**No**	**Yes**	**Intracellular**
**c.1132C>T**	**p.Pro378Ser (P378S)**	**8**	**Kinase domain**	**-**	**No**	**Yes**	**Intracellular**
c.1134C>T	p.Pro378Pro (P378P)	8	Kinase domain	Yes	Yes	Yes	Cell surface
**c.1135G>A**	**p.Glu379Lys (E379K)**	**8**	**Kinase domain**	**-**	**No**	**Yes**	**Intracellular**
c.1137G>T	p.Glu379Asp (E379D)	8	Kinase domain	-	No	Yes	Intracellular
c.1211T>G	p.Val404Gly (V404G)	8	Kinase domain	-	No	Yes	Cell surface
**c.1231C>T**	**p.Arg411Trp (R411W)**	**8**	**Kinase domain**	**-**	**No**	**Yes**	**Cell surface**
**c.1249A>T**	**p.Ile417Phe (I417F)**	**9**	**Kinase domain**	**Yes**	**Yes**	**Yes**	**Cell surface**

Analogously, Klaus et al. found that p.Cys77Trp, was expressed, but it did not reach the cell surface properly, did not bind BMP9 and did not respond to BMP9 [44]. These characteristics are in line with our observations concerning p.Cys77Phe. This mutant, while it is expressed, did not reach the cell surface and did not respond to BMP9. These cysteine substitutions disrupt the fourth disulfide bridge [1]. The residue Arg411 is conserved in the TGF-β type I receptor family [37]. The p.Arg411Trp has been described and was found defective in response to BMP9 [1] in accordance with our results. This arginine has been also described as mutated to glutamine and proline and both mutants did not respond to BMP9 indicating that this amino acid is essential for ALK1 activity [1] [37] [45].

Concerning p.Glu111Asp, p.Ile245Val, p.Pro378Pro and p.Ile417Phe, these 4 mutations have responded to the BMP9 stimulation, were well folded as revealed by western blot, and were addressed to the plasma membrane. The substitution c.1134C>T (p.Pro378Pro), is a rare polymorphism. It was found in a patient, who carries a deletion in the *ACVRL1* which is the cause of the disease. We have controlled that the splicing of a minigene bearing this polymorphism was identical to the one observed with the wild type construct. This silent mutation was introduced to give another positive control to the activity analysis. The patient carrying the mutation c.333G>C (p.Glu111Asp) was found to also carry an *ENG* mutation affecting the first methionin (p.Met1Ile), which is probably the cause of the pathology. We suggest that p.Glu111Asp is unlikely to cause HHT disease but instead represents a rare polymorphism. As for the substitution c.1134C>T, we controlled by minigene assay that the splicing was identical to the wild type construct. Furthermore, the p.Glu111Asp mutation was later identified in the proband’s father who showed no symptoms of HHT, then giving more evidence to our result. Moreover, very recently, we investigated the two children of this patent, both showing symptoms of HTT and both carrying the pathogenic mutation (p.Met1Ile), but only one carrying the p.Glu111Asp variation. For c.1249A>T (p.Ile417Phe), the patient has a mutation in exon 2 of ENG gene (c.132_133delTA; p.Tyr44fs46Ter). The position of the point mutation at the beginning of exon 9 led us to test it by Splicing minigene and results showed no effect on mRNA maturation of exon 9. Thus, this result, added to those of the functional test and protein expression, suggests that this variant is also a rare polymorphism.

Patient with c.733A>G (p.Ile245Val) have no other mutations neither in *ACVRL1* nor *ENG*. Since this mutation is of missense type, it may potentially affect the protein structure impairing partially or totally the receptor activity. According to this hypothesis, we first assayed the response to BMP9 to preliminary evaluate the functional activity. Our result showed a normal activity.

Nevertheless, the literature describes an increasing number of human diseases resulting from aberrant splice site selection [46]. Yet, many previous studies demonstrated that synonymous mutations and SNPs can have a strong effect on alternative splicing and disease outcome [47, 48]. Based on these advances, we hypothesized that the nucleotide mutation in exon 6, c.733G>A (p.Ile245Val) could have an effect on pre-mRNA splicing. The rapid cloning of PCR products using pSpliceExpress has greatly facilitated the investigation aimed to determine such changes. Our RT-PCR data reveals that c.733A>G induces the synthesis of an mRNA missing 40bp of the exon 6 compared to the normal allele, as it was predicted by bioinformatic splicing tools. This novel mRNA exhibits a PTC in the exon 7 and it will be degraded by NMD. Our results confirm that some exonic mutations are underestimated in the regulation of the pre-mRNA splicing. Conceivably, it was difficult to obtain RNA from patient to analyze the expression level of *ACVRL1* mutant alleles and to confirm the involvement of the NMD mechanism in the modulation of ALK1 receptor expression. The *in vivo* studies by an overlapping RT-PCR and sequencing of the coding region of the *ACVRL1* gene in probands should be performed to provide more confidence in such splicing analysis.

For one patient, we found an intronic mutation in the flanking region of exon 7 of *ACVRL1*: c.1048+5G>A. To characterize the functional significance of this point mutation, we used the reporter minigene technique to analyze a splicing defect in the exon-intron boundaries of the exon 7. RT-PCR showed that the c.1048+5G>A activates a cryptic acceptor splice site, as predicted by algorithm analysis, generating a transcript including 35bp of the intron 7. This intronic inclusion induces a frameshift and a premature stop codon appears at the beginning of the exon 8, probably resulting in the pre-mRNA degradation by NMD mechanism.

Our work provides molecular evidence about the pathogenicity of 23 mutations in *ACVRL1*. For two single cases, we demonstrated that the missense as well as the intronic mutations can have an impact on mRNA splicing rather than on the protein structure. As conclusion, our reported findings agree with the functional haploinsufficiency model of HHT2 at three levels; an aberrant splicing as the primary pathomechanism leading to NMD-mediated mRNA degradation, an ER sequestration of a misfolded mutated protein reducing the protein expression at the membrane, and an inactive receptor impaired in its enzymatic activity or its ligand affinity. Finally, the methodological approach used is this work has proved to be an useful tool for verifying the pathogenicity of *ACVRL1* mutations.

## Supporting Information

S1 Fig Subcellular localization of ALK1 protein variants. Known mutations are represented in S1.A and S1.B and novel mutations are represented in S1.C, S1.D and S1.E. Hela cells were transiently transfected with the C-terminally HA-tagged ALK1 plasmids. Subsequently the permeabilised cells were incubated with mouse monoclonal anti- HA antibody and co-stained with rabbit polyclonal anti- calnexin antibody and then processed for fluorescence confocal microscopy. For 22 cases, the mutants (I417F, Q111D, I245V, and P378P) reached the cell surface similarly to the ALK1-Wt and the remaining mutant proteins (R47P, G211D, L306P, H314Y, P378S, E379K, R411W, C41G, C46G, H66Y, C77F, G211S, L313V, E379D, V404G, V441M and C443Y) co-localized with the ER marker and showed an impairment of plasma membrane localization of the ALK1 receptors. C41Y, L313V and V404G mutants can reach the plasma membrane but their localization in the ER network was predominant. Bars = 5μm.
(TIF)

S1 Table Primers sequences used for site-direct mutagenesis to generate the 14 novel mutations in S1APrimers sequences used for site-direct mutagenesis to generate the 8 known mutations in S1B. (DOCX)

S2 Table Primers sequences used for generation of minigene reporters. (DOCX)
