# Supplementary figures and images for: Functional and splicing defect analysis of 23 ACVRL1 mutations in a cohort of patients affected by Hereditary Hemorrhagic Telangiectasia
Source: PLoS One. 2015 Jul 15;10(7):e0132111. doi: 10.1371/journal.pone.0132111 (PMC4503601; doi:10.1371/journal.pone.0132111)

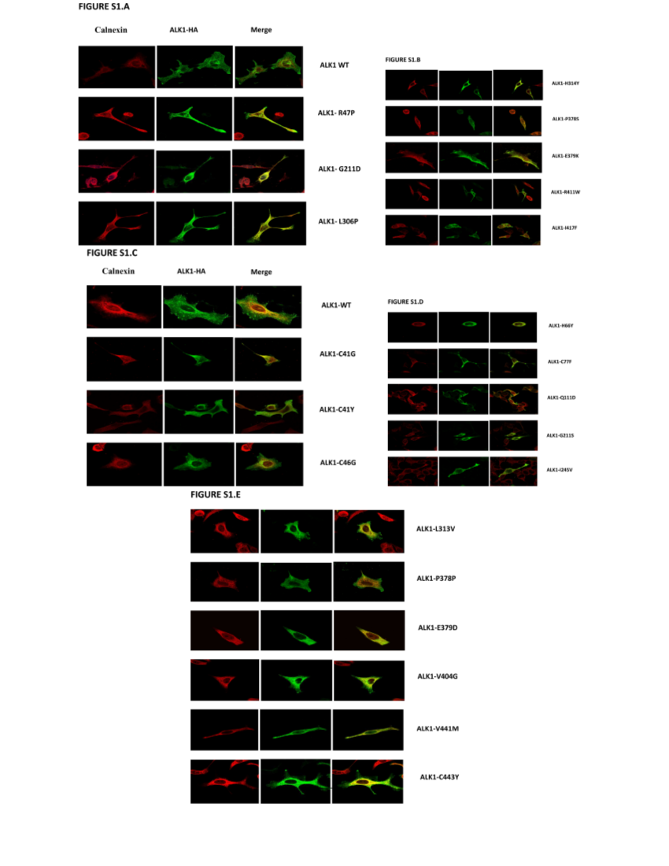

Supplement: S1 Fig — Known mutations are represented in S1.A and S1.B and novel mutations are represented in S1.C, S1.D and S1.E. Hela cells were transiently transfected with the C-terminally HA-tagged ALK1 plasmids. Subsequently the permeabilised cells were incubated with mouse monoclonal anti- HA antibody and co-stained with rabbit polyclonal anti- calnexin antibody and then processed for fluorescence confocal microscopy. For 22 cases, the mutants (I417F, Q111D, I245V, and P378P) reached the cell surface similarly to the ALK1-Wt and the remaining mutant proteins (R47P, G211D, L306P, H314Y, P378S, E379K, R411W, C41G, C46G, H66Y, C77F, G211S, L313V, E379D, V404G, V441M and C443Y) co-localized with the ER marker and showed an impairment of plasma membrane localization of the ALK1 receptors. C41Y, L313V and V404G mutants can reach the plasma membrane but their localization in the ER network was predominant. Bars = 5μm. (TIF) [file pone.0132111.s001.tif]
