# Supplementary material for: Functional and splicing defect analysis of 23 ACVRL1 mutations in a cohort of patients affected by Hereditary Hemorrhagic Telangiectasia
Source: PLoS One. 2015 Jul 15;10(7):e0132111. doi: 10.1371/journal.pone.0132111 (PMC4503601; doi:10.1371/journal.pone.0132111)
Supplement: S1 Table — (DOCX) [file pone.0132111.s002.docx]

| MUT121F | GTGTGAGAGCCCACATGGCAAGGGGCCTACCTG |
| --- | --- |
| MUT121R | CAGGTAGGCCCCTTGCCATGTGGGCTCTCACAC |
| MUT122F | GTGTGAGAGCCCACATTACAAGGGGCCTACCTG |
| MUT122R | CAGGTAGGCCCCTTGTAATGTGGGCTCTCACAC |
| MUT136F | GCAAGGGGCCTACCGGCCGGGGGGCCTGG |
| MUT136R | CCAGGCCCCCCGGCCGGTAGGCCCCTTGC |
| MUT196F | GGAGGCACCCCCAGGAATATCGGGGCTGCGGG |
| MUT196R | CCCGCAGCCCCGATATTCCTGGGGGTGCCTCC |
| MUT230F | TGCACAGGGAGCTCTTCAGGGGGCGCCCCACC |
| MUT230R | GGTGGGGCGCCCCCTGAAGAGCTCCCTGTGCA |
| MUT333F | CCCAACCTCCTTCGGACCAGCCGGGAACAGATGG |
| MUT333R | CCATCTGTTCCCGGCTGGTCCGAAGGAGGTTGGG |
| MUT631F | GAGTGTGTGGGAAAAAGCCGCTATGGCGAAG |
| MUT631R | CTTCGCCATAGCGGCTTTTTCCCACACACTC |
| MUT733F | GGTTCCGGGAGACTGAGGTCTATAACACAGTGTTGC |
| MUT733R | GCAACACTGTGTTATAGACCTCAGTCTCCCGGAACC |
| MUT937F | ATGCGGCCTGGCGCACGTGCACGTGGAGATCTT |
| MUT937R | AAGATCTCCACGTGCACGTGCGCCAGGCCGCAT |
| MUT1134F | GCGGTACATGGCACCTGAGGTGCTGGACGAGC |
| MUT1134R | GCTCGTCCAGCACCTCAGGTGCCATGTACCGC |
| MUT1137F | GGTACATGGCACCCGATGTGCTGGACGAGCAG |
| MUT1137R | CTGCTCGTCCAGCACATCGGGTGCCATGTACC |
| MUT1211F | GGGCCTTTGGCCTGGGGCTGTGGGAGATTGC |
| MUT1211R | GCAATCTCCCACAGCCCCAGGCCAAAGGCCC |
| MUT1321F | GCTTTGAGGACATGAAGAAGATGGTGTGTGTGGATCAGC |
| MUT1321R | GCTGATCCACACACACCATCTTCTTCATGTCCTCAAAGC |
| MUT1328F | CATGAAGAAGGTGGTGTATGTGGATCAGCAGACCC |
| MUT1328R | GGGTCTGCTGATCCACATACACCACCTTCTTG |

**S1A Table.** Primers sequences used for site-direct mutagenesis to generate the 14 novel mutations

| MUT140F | GGGGCCTACCTGCCCGGGGGCCTGGTGC |
| --- | --- |
| MUT140R | GCACCAGGCCCCCGGGCAGGTAGGCCCC |
| MUT632F | GAGTGTGTGGGAAAAGACCGCTATGGCGAAG |
| MUT632R | CTTCGCCATAGCGGTCTTTTCCCACACACTC |
| MUT916F | GGCTAGCTGTGTCCCCGGCATGCGGCCTGG |
| MUT916R | CCAGGCCGCATGCCGGGGACACAGCTAGCC |
| MUT940F | GGCCTGGCGCACCTGTACGTGGAGATCTTCG |
| MUT940R | CGAAGATCTCCACGTACAGGTGCGCCAGGCC |
| MUT1132F | CCAAGCGGTACATGGCATCCGAGGTGCTGGACGAGC |
| MUT1132R | GCTCGTCCAGCACCTCGGATGCCATGTACCGCTTGG |
| MUT1135F | GCGGTACATGGCACCCAAGGTGCTGGACGAGCAG |
| MUT1135R | CTGCTCGTCCAGCACCTTGGGTGCCATGTACCGC |
| MUT1231F | GCTGTGGGAGATTGCCCGCTGGACCATCGTGAATGG |
| MUT1231R | CCATTCACGATGGTCCAGCGGGCAATCTCCCACAGC |
| MUT1249F | CGGACCATCGTGAATGGCTTCGTGGAGGACTATAGACC |
| MUT1249R | GGTCTATAGTCCTCCACGAAGCCATTCACGATGGTCCG |

**S1B Table**. Primers sequences used for site-direct mutagenesis to generate the 8 known mutations.
