# Supplementary material for: Functional and splicing defect analysis of 23 ACVRL1 mutations in a cohort of patients affected by Hereditary Hemorrhagic Telangiectasia
Source: PLoS One. 2015 Jul 15;10(7):e0132111. doi: 10.1371/journal.pone.0132111 (PMC4503601; doi:10.1371/journal.pone.0132111)
Supplement: S2 Table — (DOCX) [file pone.0132111.s003.docx]

| AttB1F | GGGGACAAGTTTCTACAAAAAAGCAGGCT’ |
| --- | --- |
| AttB2R | GGGGACCACTTTGTACAAGAAAGCTGGGT’ |
| ALK1ep6A | GGGGACAAGTTTGTACAAAAAAGCAGGCTGGCTAGGTTCTTCTTTCTGCA |
| ALK1epi6B | GGGGAC CACTTTGTACAAGAAAGCTGGGTTGACAGAGCGAGGCCCTGT |
| ALK1epi7A | GGGGACAAGTTTGTACAAAAAAGCAGGCTGTGTTGGGATTACAGGTGCAA |
| ALK1epi7B | GGGGACCACTTTGTACAAGAAAGCTGGGTGTCCTGCCTCCTTTCTCCC |
| ALK1epi9A | GGGGACAAGTTTGTACAAAAAAGCAGGCTTCTGGCACATGCCATGTGCA |
| Alk1epi9B | GGGGACCACTTTGTACAAGAAAGCTGGGTGCCACAGATAGCCTCTGACT |

**S2 Table**. Primers sequences used for generation of minigene reporters.
